# Predictive risk factors before the onset of familial rheumatoid arthritis: the Tatarstan cohort study

**DOI:** 10.3389/fmed.2023.1227786

**Published:** 2023-10-09

**Authors:** Marina I. Arleevskaya, Regina V. Larionova, Elena I. Shagimardanova, Natalia E. Gogoleva, Olga A. Kravtsova, Andrej A. Novikov, Gevorg G. Kazarian, Caroline Carlé, Yves Renaudineau

**Affiliations:** ^1^Central Research Laboratory, Kazan State Medical Academy, Kazan, Russia; ^2^Institute of Fundamental Medicine and Biology, Kazan (Volga Region) Federal University, Kazan, Russia; ^3^Innovation Department, Sobolev Institute of Mathematics, Siberian Brunch of Russian Academy of Science, Novosibirsk, Russia; ^4^Immunology Department Laboratory, Institut Fédératif de Biologie, Toulouse University Hospital Center, Toulouse, France; ^5^INFINITy, Toulouse Institute for Infectious and Inflammatory Diseases, INSERM U1291, CNRS U5051, University Toulouse III, Toulouse, France

**Keywords:** familial rheumatoid arthritis, risk factors, shared epitope, low education, symmetrical arthralgia, childless

## Abstract

**Background:**

A familial history of rheumatoid arthritis (RA) predisposes an individual to develop RA. This study aimed at investigating factors associated with this conversion from the Tatarstan cohort.

**Methods:**

A total of 144 individuals, referred to as pre-RA and at risk for familial RA, were selected 2 years (range: 2–21 years) before conversion to RA and compared to non-converted 328 first-degree relatives (FDR) from RA as assessed after ≥2 years follow-up, and 355 healthy controls were also selected (HC). Preclinical parameters and socio-demographic/individual/HLA genetic factors were analyzed when data were available at the time of enrollment.

**Results:**

As compared to FDR and HC groups, pre-RA individuals were characterized before conversion to RA by the presence of arthralgia, severe morning symptoms, a lower educational level, and rural location. An association with the HLA-DRB1 SE risk factor was also retrieved with symmetrical arthralgia and passive smoking. On the contrary, alcohol consumption and childlessness in women were protective and associated with the HLA-DRB1^*^07:01 locus.

**Conclusion:**

Before RA onset, a combination of individual and genetic factors characterized those who are at risk of progressing to RA among those with familial RA relatives.

## 1. Introduction

Susceptibility for rheumatoid arthritis (RA) involves genetic and environmental factors explaining a familial risk (2× -5×) for developing RA as compared to the general population (1, 2). In this high-risk population for future development of RA, preclinical symptoms are frequently present including arthralgia, swelling, and morning stiffness, which are used to define clinically suspect arthralgia (CSA) (3). However, many questions remain since not all relatives of RA patients will develop RA, including those with CSA, which supports the possible involvement of additional parameters such as socio-demographic factors (e.g., age, sex, pregnancy, and education level), individual behavior factors (e.g., active/passive tobacco smoking), and genetic factors [e.g., human leukocyte antigen (HLA)].

Located in Russia at the crossroads of the East and West, the population of Tatarstan is characterized by an elevated risk for RA development among first-degree relatives (FDR) of RA patients (9.1 cases/1,000/year) (4). In a previous study, 26 FDR women having developed RA were studied at different time points showing a relationship between joint symptoms and then susceptibility to upper respiratory tract infection events in 3 years preceding the RA onset (4). In this cohort, herpes simplex virus (HSV) reactivation and oral microbiome changes also characterize the familial RA onset (5, 6). To proceed further with the analysis of this cohort, 144 RA patients with a familial RA history were included when a rheumatological evaluation and information regarding socio-demographic and individual behavior factors were recorded at least 2 years before the RA onset. Biological factors were tested *a posteriori* from available samples. Next, pre-RA individuals with familial RA were compared with FDR without RA evolution and healthy controls (HC).

## 2. Materials and methods

### 2.1. Subjects

From its initiation in 1997 and as previously described (4), a rheumatological pre-clinical evaluation and a questionnaire regarding socio-demographic and individual behavior factors are proposed to FDR from RA patients (i.e., kids, siblings, and parents), and such evaluation has been extended to second-degree relatives (SDR: grandparents, aunts, uncles, nieces, and nephews) in multicase RA families. Among them, 144 pre-RA individuals were selected based on two criteria: (i) first, fulfilling the 2010 American College of Rheumatology (ACR)/European League Against Rheumatism (EULAR) criteria at diagnosis (7), and before 2010, a consensus diagnosis was made by three experienced rheumatologists; and (ii) second, an evaluation conducted ≥ 2 years before the onset of RA.

Control groups were composed of familial RA individuals who had not progressed to RA (FDR, *n* = 328) after ≥ 2 years of follow-up and healthy controls (HC, *n* = 355). HC included subjects without any signs of chronic disease and no autoinflammatory/autoimmune diseases among first- and second-degree relatives. The study was approved by the Ethical Committee of the Kazan State Medical Academy, Kazan, Russia (Permit no. 1/2002). Consent to conduct studies and to allow publication of the results was received from all the individuals involved in the study according to the legal requirements in Russia.

### 2.2. Clinical, environmental, and serological factors

At the inclusion visit, an evaluation of joint symptoms was performed by a rheumatologist, and the evaluation was completed with magnetic resonance imaging (MRI) in the case of joint symptoms (pain and morning stiffness) in the small joints of the feet and hands. The seven criteria that define CSA were collected from medical records to evaluate CSA status (positivity ≥ 3): arthralgia (≤1 year), metacarpophalangeal (MCP) arthralgia, morning stiffness ≥ 60 min, severe symptoms present in the morning, difficulty with making a fist by testing the strength and the ability to completely close the fist, positive MCP squeeze test, and the presence of FDR with RA (3). In some cases, due to the design of the study that included RA relatives, the “presence of FDR with RA” was not included as the criterion in the CSA score, and this was specified. Before the formulation of the CSA criteria in 2017 and as presented in **Table 2**, the non-standard assessment of pre-RA activity was performed using 11 criteria that were used a posteriori to evaluate CSA status or were referred to as non-CSA criteria to be included in the analysis (small/large/upper and lower/symmetrical/lower limb arthralgia) (8–12). Moreover, parameters collected at the inclusion included socio-demographic factors (age, sex, rural residence, childlessness, and education level) and individual behavior factors (fish/alcohol/coffee consumption and active/passive/no tobacco smoking). For statistical purposes, education was dichotomized into low educational level (secondary and high school graduates) and high educational level (university graduates). When serum was available at the time of the inclusion, anti-CCP and IgM RF were tested.

### 2.3. Genetic factors

HLA typing was performed by DNA sequencing of loci A, B, C, and DQB1 and 2–4 exons of the DRB1 gene using the HLA holotype kit (Omixon, Budapest, Hungary). Briefly, the isolated DNA concentration was estimated using an Implen NP80 NanoPhotomer (Fisher Scientific, Pittsburgh, PA), and long-range PCR was performed next with the resulting amplicons of the five loci. Then, in the process of library preparation, the amplicon pools were fragmented, ends were repaired, and barcodes were ligated. Finally, the sequencing was performed on a MiSeq next-generation sequencing (NGS; Illumina, San Diego, CA) instrument in the pair-terminal reading mode. The readings obtained (251 pb) were analyzed using the HLA twin software (Omixon) with two algorithms in a day-by-day assembly mode and by mapping against the global international ImMunoGeneTics (IMGT) database. The following alleles were considered as shared epitope (SE) alleles: DRB1^*^01:01, ^*^01:02, ^*^04:01, ^*^04:04, ^*^04:05, ^*^04:08, and ^*^10:01 (13).

### 2.4. Statistics

Quantitative results are expressed as the mean and interquartile (IQ) and compared by pairwise analysis. Categorical data were analyzed using Fisher's exact test, and when appropriate, a false discovery rate *post-hoc* correction was applied, and an odds ratio (OR) with a 95% confidence interval (CI) was determined. All tests and figures were built with Prism 9.4 (GraphPad Software, La Jolla, CA), with the exception of the radar plots (Microsoft Corp, Redmond, WA).

## 3. Results

### 3.1. Population characteristics

As presented in Table 1, 827 individuals included in this study were subdivided into three groups: pre-RA stage corresponding to 144 individuals having evolved to RA [median: −3 years (IQ: −2/−10 years) from diagnosis]; 328 FDR from RA and without RA evolution after ≥ 2 years follow-up; and 355 HC. Age at inclusion was similar between pre-RA and FDR, while older in HC. The three groups included more women than men, which is related to the design of the Tatarstan cohort that favors the inclusion of women. Pre-RA, FDR, and HC subgroups were similar with regard to ACPA positivity, and RF positivity was lower in HC.

**Table 1:** Demographic and clinical characteristics of the population studied.

	**Controls**	**Familial RA**	**Familial RA with RA evolution**		**Pre-RA vs. HC (*p*)**	**Pre-RA vs. FDR (*p*)**
		**FDR**	**At the pre-RA stage**	**At the RA stage**		
*N*	355	328	144	144	–	–
Age, median (IQ)	50 (26–59)	39 (28–52)	40 (29–50)	47 (34–54)	0.0002	0.960
Female (%)	341 (96.1%)	320 (97.6%)	135 (94.4%)	135 (94.4%)	0.345	0.06
FDR/SDR/no	0/0/355	328/0/0	105/39/0	105/39/0	<10^−4^	<10^−4^
Time to RA (IQ)	–	–	−2.0 y (−2.0/−6.0)	1.6 y (0.0–10)	–	–
DAS28-ESR (IQ), *n*	–	–	–	5.1 (4.3–6.1), *n* = 95	–	–
HAQ (IQ), *n*	–	–	–	1.7 (0.9–3.0), *n* = 109	–	–
ESR ≥ 30 mm/h (%)	–	–	–	64/110 (58.2%)	–	–
CRP ≥ 5 mg/L (%)	–	–	–	71/104 (68.3%)	–	–
ACPA ≥ 20 U/mL (%)	2/51 (3.9%)	19/123 (15.4%)	3/36 (8.3%)	65/86 (75.6%)	0.384	0.346
RF ≥ 14 IU/mL (%)	4/136 (2.3%)	29/172 (16.9%)	6/50 (12%)	83/116 (71.6%)	0.02	0.354

IQ, interquartile; RA, rheumatoid arthritis; DAS28, joints 28 disease activity score; HAQ, health assessment questionnaire; ESR erythrocyte sedimentation rate; CRP, C-reactive protein; ACPA, anticitrullinated peptide antibodies; RF, rheumatoid factor.

At the RA stage, the 144 pre-RA individuals presented an elevated disease activity score (DAS28-ESR, median = 5.1), a functional impact of the disease (HAQ, median = 1.7), inflammation (ESR ≥ 30 mm/h, 58.2%; and CRP ≥ 5 mg/L, 68.3%), and the detection of ACPA (≥20 U/mL, 75.6%) and/or RF (≥14 IU/mL, 71.6%).

### 3.2. Clinical risk factors in pre-RA

At the early phase of RA (3, 14), a CSA score of ≥3 was effective in distinguishing pre-RA from HC (*p* < 10^−4^). However, to distinguish pre-RA from FDR, the criteria “presence of FDR with RA” had to be removed from the CSA score (*p* = 0.0005).

**Figure 1:**
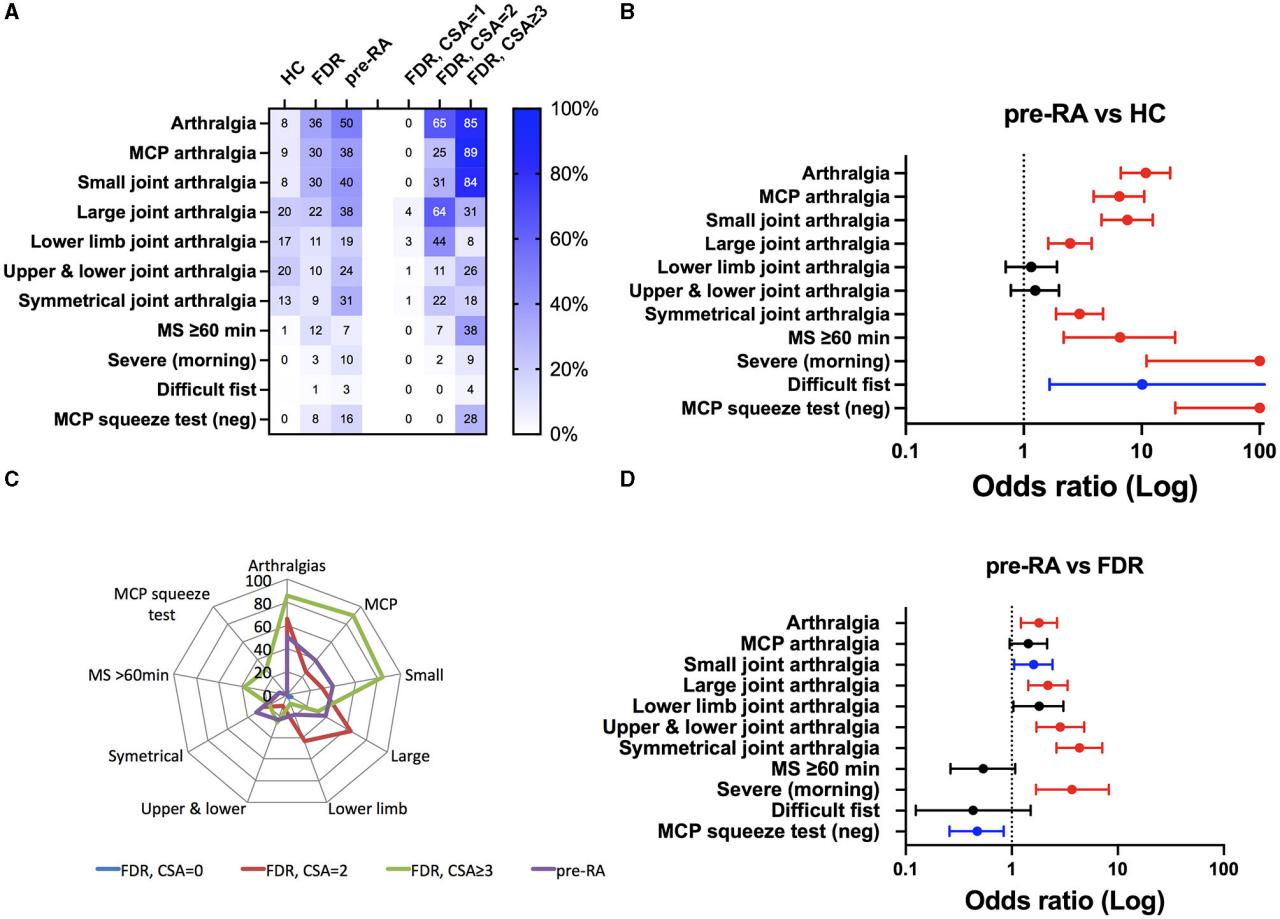
Clinical spectrum at the inclusion visit in the Tatarstan cohort of healthy controls (HC, *n* = 355) and rheumatoid arthritis (RA) relatives. Later being dichotomized according to their progression into RA (pre-RA, *n* = 144) or as FDR when no evolution was reported during the ≥2 years follow-up. **(A)** Prevalence of the 11 RA-associated clinical criteria, see Section 2 for details. FDR individuals were further subdivided according to their clinically suspected arthralgia (CSA) score. **(B, C)** odds ratio (OR) and significant *p*-values (in blue when 0.01 < *p* < 0.05, and in red *p* < 0.01 corresponding to the *post-hoc* adapted threshold) for the occurrence of the clinical criteria when pre-RA individuals were compared to HC **(B)** or compared to FDR **(C)**. **(D)** Radar plot comparing clinical criteria between pre-RA individuals and FDR according to their CSA status.

Next, to gain further insight into the relevance of disabilities in assessing those familial RA individuals at risk of developing RA, 11 clinical parameters were selected from CSA criteria (*n* = 6) and from additional arthralgia characteristics (*n* = 5). As presented in Table 2 and Figures 1A, B, all these parameters, except isolated lower limb arthralgia and pain joints in the upper and lower extremities (black line), were effective in distinguishing pre-RA from HC with an OR ranging from 1.6 to infinity. Among them, significance was conserved when applying a *post-hoc* correction (red line) except for difficulties with making a fist (0.01 < *p* < 0.05, blue line).

**Table 2:** Characteristics of individuals at inclusion in the Tatarstan cohort.

	**HC (*n* = 355)**	**FDR (*n* = 328)**	**Pre-RA (*n* = 144)**	**Pre-RA vs. HC (*p* =)**	**Pre-RA vs. FDR (*p* =)**
CSA ≥ 3 (%)	2 (0.6%)	97 (30.0%)	53 (36.8%)	<10^−4^	0.133
CSA-FDR ≥ 3 (%)	2 (0.6%)	29 (8.8%)	30 (20.8%)	<10^−4^	5 × 10^−4^
Arthralgia	30 (8.5%)	117 (35.7%)	72 (50.0%)	<10^−4^	0.004
MCP arthralgia	31 (8.7%)	99 (30.2%)	55 (38.2%)	<10^−4^	0.089
MS (≥60 min)	4 (1.1%)	40 (12.2%)	10 (6.9%)	0.001	0.104
Severe (morning)	0	10 (3.0%)	15 (10.4%)	<10^−4^	0.003
Difficult fist	1 (0.3%)	4 (1.2%)	4 (2.8%)	0.026	0.255
MCP squeeze test	0	27 (11.3%)	23 (16.0%)	<10^−4^	0.015
Small arthralgia	29 (8.2%)	97 (29.6%)	58 (40.3%)	<10^−4^	0.026
Large arthralgia	71 (20%)	72 (22.0%)	55 (38.2%)	<10^−4^	4 × 10^−4^
Upper and lower arthralgia	70 (19.7%)	32 (9.8%)	34 (23.6%)	0.334	1 × 10^−4^
Lower limb only arthralgia	59 (16.6%)	37 (11.3%)	27 (18.8%)	0.601	0.40
Symmetrical arthralgia	47 (13.2%)	31 (9.5%)	45 (31.2%)	<10^−4^	<10^−4^
Childlessness	35/177 (19.8%)	37/116 (31.9%)	15/112 (13.4%)	0.144	4 × 10^−4^
Educational low	126/206 (61.2%)	65/146 (44.5%)	92/118 (78.0%)	0.017	<10^−4^
Rural	113/186 (60.5%)	60/149 (40.3%)	94/128 (73.4%)	<10^−4^	<10^−4^
Fish consumption	133/175 (76%)	89/124 (71.8%)	93/119 (78.2%)	0.501	0.159
Coffee consumption	79/164 (48.2%)	52/121 (43.0%)	54/118 (45.8%)	0.735	0.590
Alcohol consumption	113/178 (63.5%)	82/124 (66.1%)	57/115 (49.6%)	0.005	0.003
Smoker active	18/188 (9.6%)	6/135 (4.4%)	3/115 (2.6%)	0.022	0.618
Smoker passive	51/188 (27.1%)	31/135 (23.0%)	49/115 (42.6%)	0.146	0.03
HLA-DRB1 SE	29/78 (37.2%)	31/50 (62%)	36/59 (61.0%)	0.005	0.916
HLA-DRB1^*^07:01	30/78 (38.5%)	11/50 (22%)	8/59 (13.6%)	0.001	0.247

BMI, body mass index; MS, morning stiffness; RA, rheumatoid arthritis; FDR, first-degree relatives; SDR, second-degree relatives; CSA, clinically suspect arthralgia; MCP, metacarpophalangeal; HLA-DRB1 SE, human leukocyte antigen alleles encoding the shared epitope; pre-RA, individuals with RA relatives having evolved to RA; FDR, relatives not having evolved to RA; wo, without.

The same analysis performed between pre-RA and FDR (Figure 1C) retrieved three groups of parameters: (i) those parameters that are ineffective in discriminating pre-RA from FDR (black line), which included MCP/lower limb arthralgia, morning stiffness of ≥60 min, and difficulty with making a fist; (ii) those parameters with low discriminating efficacy and not confirmed following *post-hoc* adjustment (0.01 < *p* < 0.05, blue line) such as small joints and a negative squeeze test in MCP joints that reflects local inflammation; and (iii) those parameters that are highly effective after *post-hoc* adjustment (red line) in discrimination including arthralgia (OR = 1.8, CI: 1.2–2.7), large joint pain (OR = 2.2, CI: 1.4–3.4), upper and lower joint pain (OR = 2.9, CI: 1.7–4.8), symmetrical arthralgia (OR = 4.4, CI: 2.6–7.1), and most severe symptoms occurring in the morning (OR = 3.7, CI: 1.7–8.2). We conclude from such analysis, and based on the radar plot comparison that considers the FDR group according to the CSA score (Figures 1A, D), that the most discriminating clinical criteria to distinguish pre-RA from FDR was symmetrical arthralgia.

### 3.3. Environmental risk factors in pre-RA

**Figure 2:**
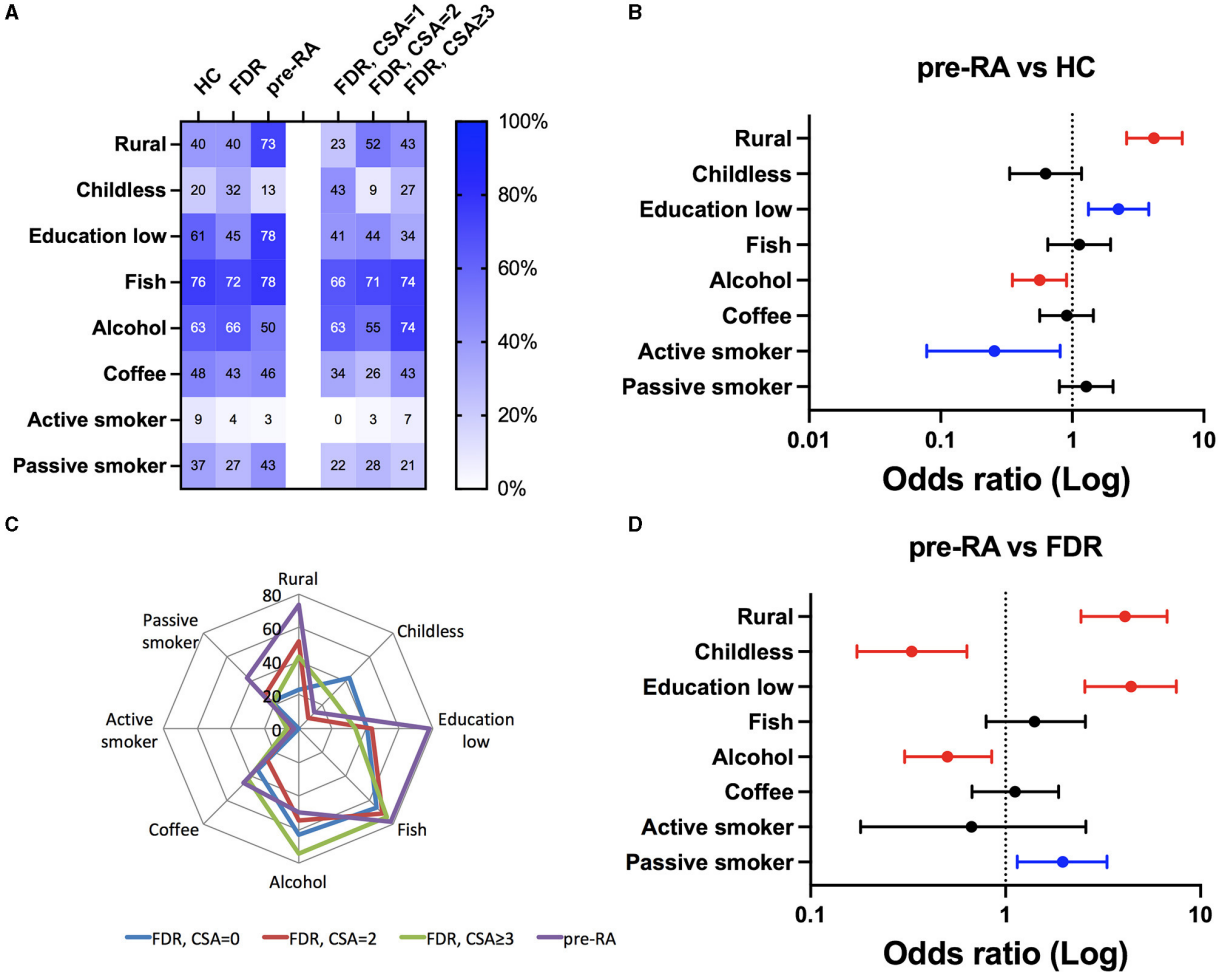
Socio-demographic and individual behavioral factors to compare healthy controls (HC) and relatives from rheumatoid arthritis (RA) patients having evolved (pre-RA) or not (FDR) to RA. **(A)** Prevalence of the eight factors in the subgroups, see Section 2 for details. FDR was further subdivided according to their clinically suspected arthralgia (CSA) score. **(B, C)** Odds ratio (OR) and significant *p*-values (in blue when 0.01 < *p* < 0.05, and in red *p* < 0.01 corresponding to the *post-hoc* adapted threshold) for the occurrence of the individual criteria when pre-RA individuals were tested against HC **(B)** or against FDR **(C)**. **(D)** Radar plot comparing individual criteria between pre-RA relatives and FDR according to their CSA status.

Epidemiological studies and more recently the use of Mendelian randomization approaches have confirmed the direct relationship between RA development and environmental factors such as educational level and tobacco smoking (2). Accordingly, socio-demographic factors (rural residence, childlessness, and educational level) and individual behavior factors (fish/alcohol/coffee consumption and active/passive tobacco smoking) were considered (Table 2; Figure 2A). Of note, the rate of inclusion for environmental risk factors ranged from 77.8 to 88.9% in pre-RA, 35.4 to 45.4% in FDR, and 49.9 to 58.0% in HC. When pre-RA individuals were compared to HC (0.01 < *p*, red line), the risk of RA development was higher among those living in rural areas (OR = 4.2; CI: 2.6–6.9) and lower in those reporting alcohol consumption (OR = 0.6, CI: 0.35–0.90) (Figure 2B).

When replacing HC with FDR in the analysis (Figure 2C), three risk factors were associated with pre-RA individuals: living in a rural environment (OR = 4.1, CI: 2.4–6.7), a lower educational status (OR = 4.4, CI: 2.6–7.5), and passive tobacco smoking (OR = 2.0, CI: 1.15–3.3). On the other hand, alcohol consumption (OR = 0.5, CI: 0.30–0.85) and childlessness (OR = 0.33, CI: 0.17–0.63) were protective, and no associations were retrieved when considering active tobacco usage and fish/coffee consumption. Among these factors, the radar plot analysis further supports prominent roles for educational attainment, rural location, and passive tobacco usage to discriminate pre-RA from FDR (Figure 2D).

### 3.4. HLA-DRB1 genetic factors in pre-RA

**Figure 3:**
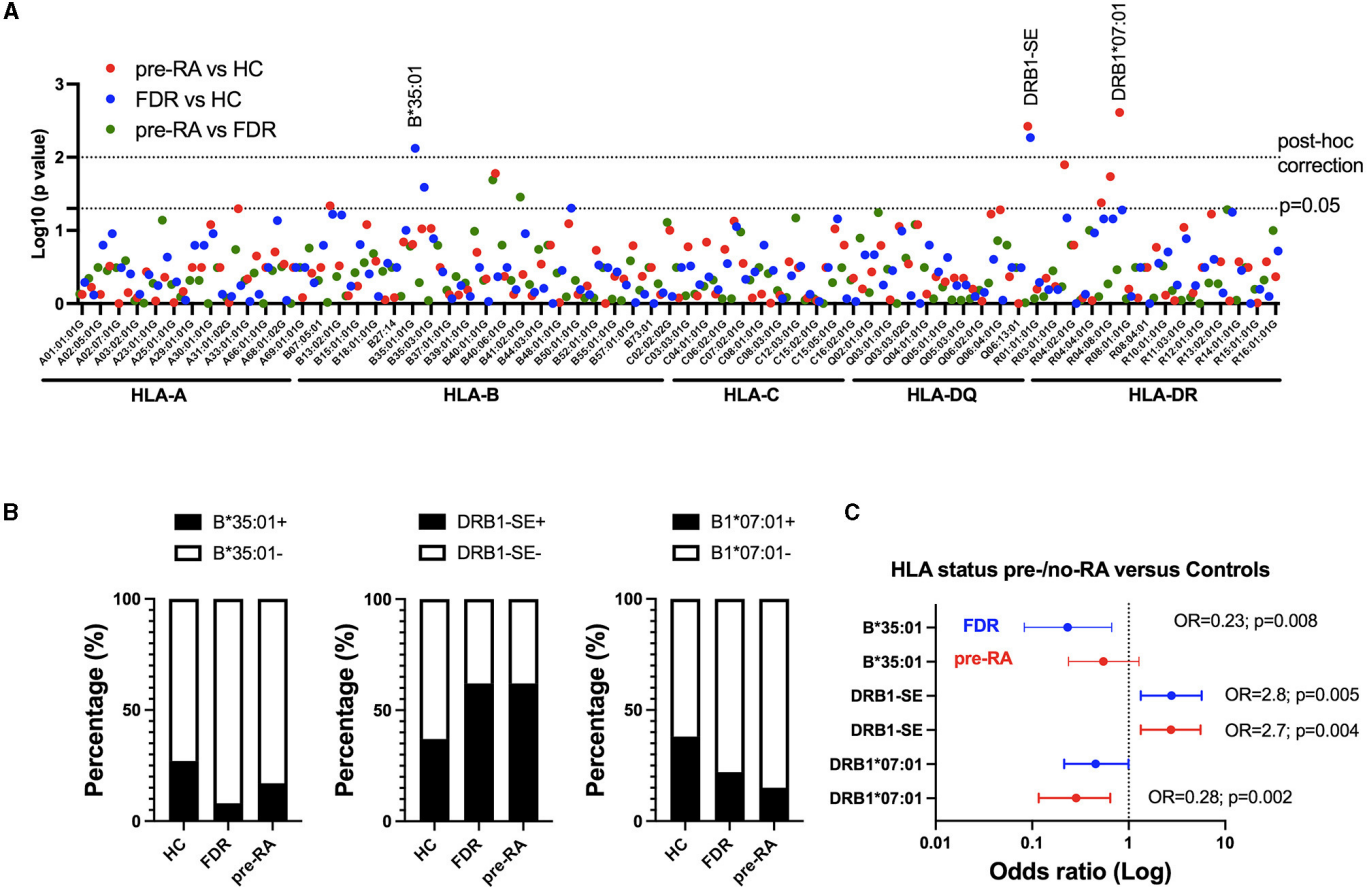
HLA A/B/C/DQB1 and DRB1 allele distribution among relatives from rheumatoid arthritis (RA) patients having evolved to RA (pre-RA) or not (FDR), and from healthy controls (HC). **(A)** Manhattan plot for the log10 (*p*-value) for each HLA allele and HLA-DQRB1 shared epitope (SE) between pre-RA and controls (red), FDR and controls (blue), and pre-RA and FDR (green). The dotted lines represent the significant *p*-values using an adapted threshold with a *post-hoc* false discovery rate approach (*p* = 0.01) or not (*p* = 0.05). **(B)** Allele frequency of HLA-B*35:01, HLA-DRB1 SE, and HLA-DRB1*07:01 among HC, FDR, and pre-RA individuals. **(C)** Odds ratio (OR) showing the genetic effect of HLA-B*35:01, HLA-DRB1 SE, and HLA-DRB1*07:01 between HC and pre-RA (red) or FDR (blue). The *p*-values are indicated when significant.

High-resolution HLA allele class I (A, B, and C) and class II (DQB1 and DRB1) distribution was assessed by NGS in pre-RA (*n* = 59), in FDR (*n* = 50), and HC (*n* = 78) in order to perform association studies between (i) pre-RA vs. HC; (ii) FDR vs. HC; and (iii) pre-RA vs. FDR (Figure 3).

Alleles with a conserved sequence at amino-acid residues 70–74 in the third hypervariable region of HLA-DRB1 and referred to as SE showed a significant association with both pre-RA individuals (OR = 2.7, CI: 1.3–5.6; *p* = 0.005) and FDR (OR = 2.8, CI: 1.3–5.7; *p* = 0.005) when compared to HC. On the other hand, a significant negative association was observed with DRB1^*^07:01 when comparing pre-RA relatives with HC (OR = 0.28, CI: 0.12–0.64; *p* = 0.002) and with B^*^35:01 when comparing FDR with HC (OR = 0.23, CI: 0.08–0.67; *p* = 0.008). As HLA B^*^35:01 was retrieved associated with FDR but not with pre-RA individuals, this allele was not considered further.

### 3.5. Genetic and environmental factors that characterize pre-RA individuals

**Figure 4:**
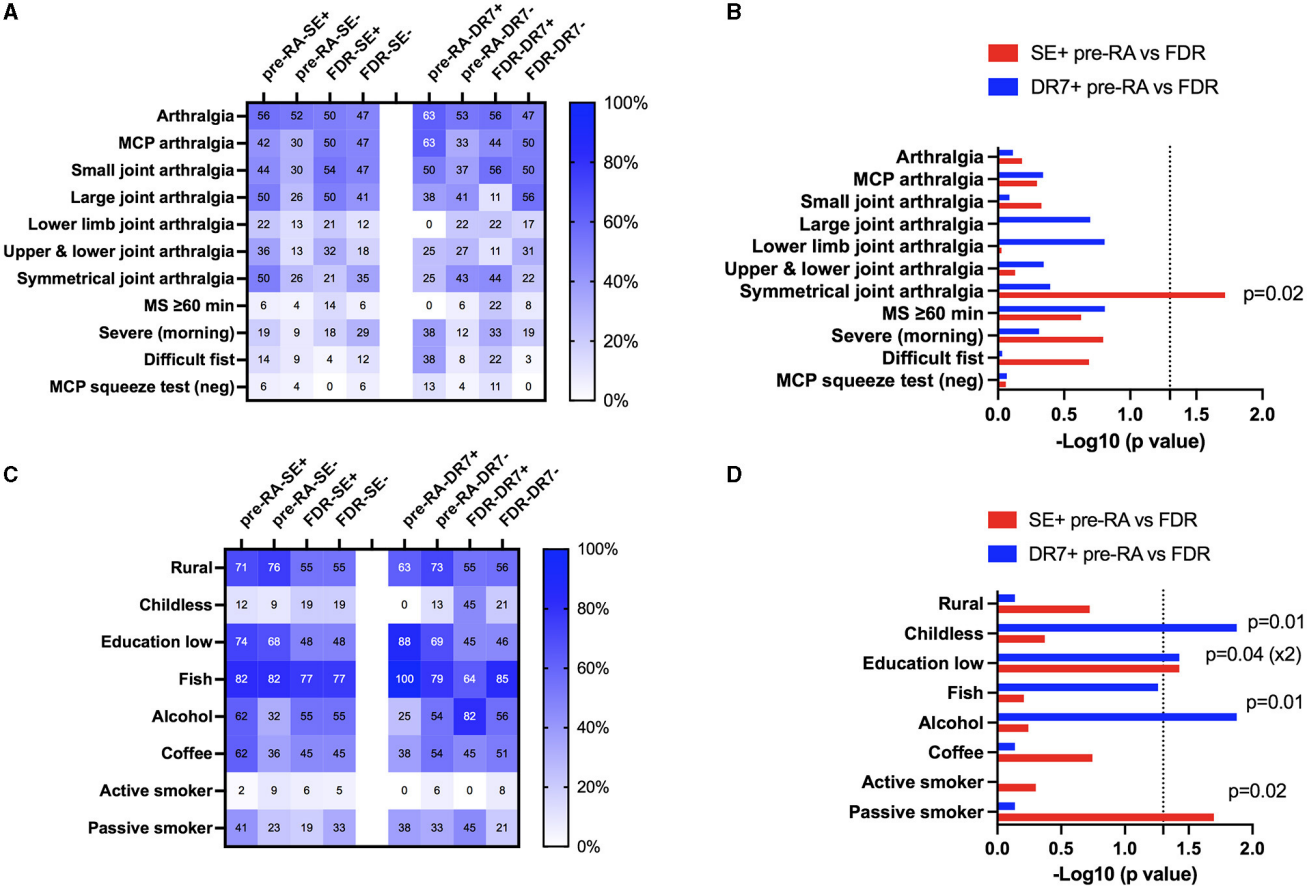
Interplay between the HLA-DRB1 shared epitope (SE) and HLA-DRB1*07:01 genetic factors and rheumatoid arthritis (RA)-associated factors in relatives from RA patients according to their evolution to RA (pre-RA) or not (FDR). **(A)** Prevalence of the 11 RA-associated clinical criteria according to their HLA-DRB1 shared epitope (SE) and HLA-DRB1*07:01 status in pre-RA and FDR. **(B)** HLA-DRB1 SE (red) and HLA-DRB1*07:01 (blue) influenced eight RA-associated risk factors to discriminate pre-RA from FDR. Data are represented as log10 (*p*-value) with a significant threshold fixed at *p* = 0.05. **(C)** Prevalence of the RA-associated individual factors. **(D)** HLA-DRB1 SE (red) and HLA-DRB1*07:01 (blue) influenced RA-associated individual factors to discriminate pre-RA from FDR.

Finally, to test whether or not clinical and individual RA-associated parameters may be influenced by HLA-DRB1-SE and HLA-DRB1^*^07:01, the comparison between pre-RA and FDR was repeated based on the HLA-DRB1 status (Figure 4). Results from such analysis revealed that symmetrical arthralgia (OR = 2.8, CI: 1.2–6.6; *p* = 0.02) and passive exposure to tobacco smoking (OR = 3.6; CI: 1.18–10.5; *p* = 0.02) predominated in pre-RA individuals harboring HLA-DRB1-SE, while alcohol consumption (OR = 0.07, CI: 0.01–0.6; *p* = 0.01) and childlessness (OR = 0.0, CI: 0–0.59; *p* = 0.01) were protective factors associated with HLA-DRB1^*^07:01. The education level attained was retrieved close to the limit of significance for both HLA-DRB1-SE (OR = 2.5, CI: 1.1–5.7; *p* = 0.04) and HLA-DRB1^*^07:01 (OR = 9.8, CI: 1.1–123; *p* = 0.04), which suggests that the education level represents an independent factor from HLA-DRB1 status.

## 4. Discussion

Results from our study support the concept that RA development in individuals with familial RA case(s) is associated with a CSA score of ≥3 without including as criteria the presence of FDR and is driven/controlled by the interplay between environmental/behavioral factors (e.g., educational level, childlessness, alcohol consumption, and passive tobacco smoking) and genetic factors (e.g., HLA-DRB1 SE and ^*^07:01).

The CSA score to identify individuals at the pre-clinical RA stage was established by the EULAR using a longitudinal study of 2 years (15). From such an analysis, it was reported that individuals meeting ≥ 3 parameters have a 2.1× increased risk of developing RA. However, such an assertion was not confirmed in our high-risk cohort characterized by a long delay (≥2 years) to develop RA within pre-RA. Relatives from familial RA with a CSA score of ≥3 at the baseline were 36.8% in pre-RA vs. 30.0% in FDR. However, when the criteria “presence of FDR with RA” was not considered, the CSA score became effective in discriminating pre-RA from FDR (20.8 vs. 8.8%, respectively). One explanation for these discrepancies is related to the fact that four out of seven criteria from the CSA score (morning stiffness of >60 min, most severe symptoms in the morning, difficulty in making a fist, and a positive squeeze test in MCP) were rarely reported in our pre-RA group (2.8–16%) as compared to the cohorts used to define CSA (43–90%) (3). Indeed, the two most prevalent symptoms retrieved in our study were arthralgia and MCP arthralgia within pre-RA and FDR groups. Moreover, and similar to our study, it was previously reported that arthralgia was higher in FDR than in HC (16), that joint symmetry was at high risk among arthralgia in FDR from RA patients (17), and that symmetry takes place as the disease progresses and may be absent at the RA onset (18).

Education attainment represents an important health and social determinant associated positively or negatively with a large panel of mental and somatic diseases (19). Regarding RA, a preventive effect is repeatedly associated with a higher educational level, and such an effect is conserved following adjustment with tobacco smoking, body mass index, and intelligence (20, 21). Indeed, in patients with RA, a higher educational level was associated with a better status including at the baseline a lower number of painful joints, less inflammation, RF seronegativity, and a higher rate of survival for men (22–24). Our study further provides arguments to support that the negative effect of education attainment on RA development starts early and is independent of HLA status. However, more studies are necessary to better characterize the exact mechanism by which education attainment controls RA development.

The HLA-DRB1 SE overrepresentation in RA was historically defined through the demonstration that the presence of HLA-DRB1 risk alleles harboring SE leads to the non-proliferative state in co-cultured lymphocytes from RA patients, which was not the case when using lymphocytes from healthy subjects (25–28). Next, it was reported that HLA-DRB1 SE represented the most predominant genetic risk factor associated with RA (29), and this overrepresentation was observed in 55% of the FDR from RA patients as compared to 43% in the HC group from North America (30). This is close to our report with 61% in pre-RA and 62% in FDR as compared to 37.2% in the HC population. Moreover, it was previously established that associations between HLA-DRB1 SE and tobacco smoking appeared at the CSA stage, while associations with autoantibody positivity (ACPA, RF) and severe disease appeared later at the RA onset, which is suggested when FDR from RA patients and early-RA patients are studied (31–33). Regarding our study, a slight association with passive tobacco smoking among pre-RA individuals carrying HLA-DRB1 SE was observed, and the absence of association with active smoking can be explained by low tobacco usage in the studied population (<10%). Regarding the association with autoantibodies (ACPA and RF), these parameters were not taken into consideration in this study as the rate of positivity in pre-RA individuals was low at inclusion (ACPA: 8.3% and RF: 12%) and not different from FDR. Moreover, HLA-DRB1 SE status was further associated with symmetrical arthralgia (OR = 2.8) when comparing pre-RA with FDR, such associations make sense and open new perspectives as joint symptoms represent key criteria in seropositive RA under progression (17, 34).

The protective role of HLA DRB1^*^07:01 was previously reported in RA (35, 36). Our study further supports an interplay between HLA DRB1^*^07:01 and the immunomodulatory effect associated with alcohol consumption and childlessness in women. The paradoxical and beneficial effect of alcohol on RA was recently reviewed as well as its mechanism of action on the innate and adaptive immune systems that have predominant roles in RA (37, 38). The relationship between childbirth and the risk of RA remains debated in the literature although it is well-established that RA often improves during pregnancy and flares can occur postpartum (39). Accordingly, part of these discrepancies regarding the contribution of pregnancy in RA development in the literature may be in relation to HLA DRB1-07:01 genetic status and/or individual behavior factors such as tobacco smoking and alcohol consumption, which require more exploration.

The main limitations of this study are its retrospective design with incomplete data sets and a database initiated before the CSA score was created. In particular, factors with a low rate of inclusion (<50%) have to be considered with caution. On the other hand, advantages are related to the homogenous genetic background reported between pre-RA and FDR individuals, the elevated number of pre-RA individuals included, and the delay of ≥2 years in the follow-up to exclude the pre-RA stage in both pre-RA and FDR groups. However, we could not firmly exclude that some individuals from the FDR subgroup would evolve to RA and that environmental risk factors have changed from inclusion to RA development such as childlessness and rural location.

In conclusion, a set of clinical, individual, and genetic characteristics to predict RA development in relatives from familial RA was established. More studies are required to determine the predictive accuracy of these parameters when used alone or when combined with autoantibody testing and/or imaging.

## Data availability statement

The data presented in this study have been uploaded to Zenodo: https://zenodo.org/record/8395440.

## Ethics statement

The studies involving human participants were reviewed and approved by Ethical Committee of the Kazan State Medical Academy, Kazan, Russia (Permit no. 1/2002). Written informed consent for participation was obtained for this study in accordance with the national legislation and the institutional requirements. Written informed consent was obtained from all the individuals involved in the study for the publication of this article.

## Author contributions

MA and YR: conceptualization, project administration, data curation, resources, formal analysis, methodology, and writing—original draft, review and editing. RL, ES, NG, OK, and GK: investigation and review and editing. AN and CC: formal analysis and review and editing. All authors contributed to the article and approved the submitted version.
